# Supplementary material for: Identification of photoperiod-induced specific miRNAs in the adrenal glands of Sunite sheep (Ovis aries)
Source: Front Vet Sci. 2022 Jul 22;9:888207. doi: 10.3389/fvets.2022.888207 (PMC9354845; doi:10.3389/fvets.2022.888207)
Supplement: Supplementary file 1 [file Data_Sheet_1.ZIP › Supplementary file/miRDeep2 file.zip!/miRDeep2 file/Novel_Structure/SP42A3/Novel_138.pdf]

Provisional ID	:	NC_019462.2_11435
Score total	:	1.5
Score for star read(s)	:	-1.3
Score for read counts	:	0
Score for mfe	:	1.2
Score for randfold	:	1.6
Score for cons. seed	:	
Total read count	:	36
Mature read count	:	36
Loop read count	:	0
Star read count	:	0

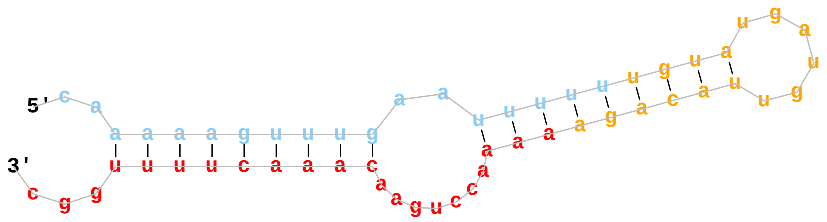

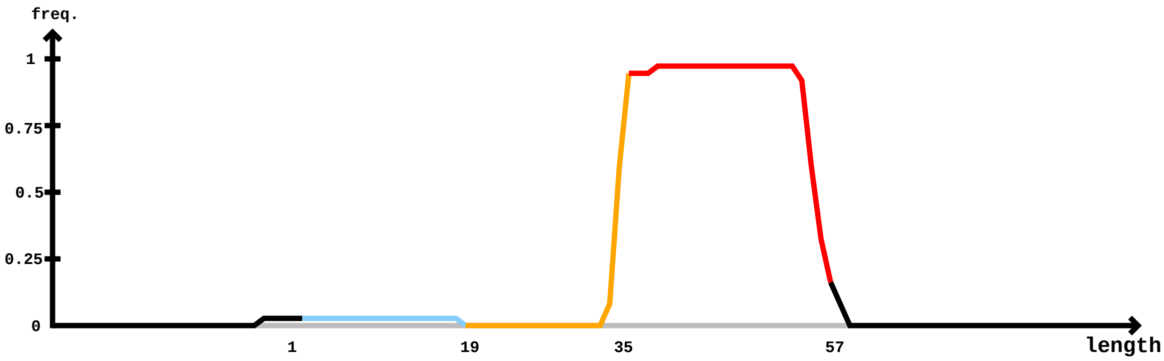

Star Mature

```
5'- ucucuggucauguauuggauuggccaaaaaguuugaauuuuuuguaugauguuacagaaaaaccugaacaaacuuuuggccaacccaauaguaauuugaaaugggcagcag -3'    exp
    .(((((.((((.((((((.((((((((((.(((((..(((((((((......)))))))))......))))))))))))))).)))))).............)))).))).))    reads    mm    sample
    ....................uggccaaaaaguuugaauuuu......................................................................    1        0     seq
    ........................................................gaaaaaccugaGcaaacuuu...................................    1        1     seq
    ........................................................gaaaaaccugaGcaaacuuuug.................................    2        1     seq
    .........................................................aaaaaccugaacaaacuuuu..................................    4        0     seq
    .........................................................aaaaaccugaacGaacuuuu..................................    4        1     seq
    .........................................................Gaaaaccugaacaaacuuuu..................................    1        1     seq
    .........................................................aaaaaccugaacaaacuuuuU.................................    3        1     seq
    .........................................................aaaaaccugaGcaaacuuuug.................................    4        1     seq
    .........................................................Gaaaaccugaacaaacuuuug.................................    1        1     seq
    .........................................................aaaaaccugaGcaaacuuuugg................................    2        1     seq
    ..........................................................aaaaccugaacaaacuuu...................................    1        0     seq
    ..........................................................aaaaUcugaacaaacuuuu..................................    1        1     seq
    ..........................................................aaaaccugaacUaacuuuu..................................    2        1     seq
    ..........................................................aaaaccugaacaaacuuuugA................................    1        1     seq
    ..........................................................aaaaccugaacaaacuuuuUg................................    3        1     seq
    ..........................................................aaaaccugaacaaacuuuuggG...............................    5        1     seq
    .............................................................aUcugaacaaacuuuuggcc..............................    1        1     seq
```
